# Theoretical and Empirical Foundations for a Unified Pyramid of Human Motivation

**DOI:** 10.1007/s12124-022-09700-9

**Published:** 2022-05-20

**Authors:** J. David Pincus

**Affiliations:** 1 Employee Benefit Research Institute, Washington, DC USA; 2 Research and Development Department, Leading Indicator Systems, One Franklin Street, Boston, MA 02110 USA

**Keywords:** Unified model of motivation, Spiritual motivation, Justice motivation, Moral motivation, Transcendental motivation

## Abstract

Revisions are proposed to the taxonomic model of human motivation of Forbes (*Review of General Psychology, 15*(2), 85-98, 2011) in order to incorporate a heretofore missing fourth life domain, the spiritual. The growing literature on spiritual motives is systematically reviewed in accordance with literature review standards for theory development (Templier & Paré, 2018) focusing on the objective of identifying comprehensive theoretical systems that explicitly incorporate the spiritual domain as one of a limited set of human life domains. The structure of the Forbes model is contrasted with thirteen theoretical systems that explicitly incorporate the spiritual as a fourth life domain. Consistent with the Forbes model, the spiritual domain is proposed to consist of three modes of existence (Being, Doing, Having) represented as justice motivation, moral motivation, and transcendental motivation, respectively, as well as both promotion and prevention goals within each of the three motives. Empirical evidence is reviewed in support of a revised heuristic device wherein the Spiritual domain is closely linked with the Intrapsychic and Interpersonal domains, but not the Instrumental domain, resulting in a pyramidal structure and corresponding set of testable hypotheses.

## Introduction

Forbes (2011) proposed a 9-cell taxonomy of human motivation formed by crossing three domains of human life, the Intrapsychic (within the self), the Instrumental (within the material world), and the Interpersonal (within the social world) with three states of being, Expectations (forward looking), Experiences (in the moment), and Outcomes (backward looking; see Table 1).

**Table 1:** The Forbes (2011) model of human motivation

	Life Domain		
States of Being	Intrapsychic (Self)	Instrumental (Material World)	Interpersonal (Social World)
Expectations (forward-looking)	Security	Empowerment	Belonging
Experiences (in the moment)	Identity	Engagement	Nurturance
Outcomes (backward-looking)	Mastery	Achievement	Esteem

However, there are unmistakable human motivations for which we cannot adequately account using the three life domains proposed by Forbes (2011). These include the set of universal needs that led to the establishment of formal spiritual systems in every known culture on earth. Among the human needs that resist classification in these three domains are the need for justice, fairness, and morality that are codified in rules of laws and systems of ethics. There is also principle-based spiritual quest motivation (Batson et al., 2005), which is the form of motivation most predictive of actual altruistic behavior, particularly acts of self-sacrifice in service of an ideal. This paper argues for the existence of a fourth fundamental domain of human life, namely, the spiritual domain, to adequately describe this set of needs within a taxonomy of human motivation.

### The Stubborn Ubiquity of Spiritually-Inspired Behavior

The present article adopts the widely cited definitions for key terms *spirituality* and the *sacred,* suggested by Pargament et al. (2005) the *sacred* as things perceived to be “holy, ‘set apart’ from the ordinary, and worthy of veneration and respect” (p. 668). The related concept of *spirituality* is defined by Hill and Pargament (2003) as the process of searching for the sacred.

Why should the concepts of spirituality and the sacred be relevant to motivation theory? There are sociological, historical, and psychological answers to this question. From a sociological perspective, all known human societies have spiritual beliefs and practices, and this has been a hallmark of all human societies since the earliest human cultures (Boyer, 2004). Globally, the vast majority of humans report holding such beliefs, and less than one fifth classify themselves as holding no such beliefs (Zuckerman, 2005). The vast majority of Americans attend some form of spiritual services regularly, pray daily, and believe in heaven, angels, and miracles (e.g., Pew Forum’s Religious Landscape Survey, 2008).

Both developmental and evolutionary psychologists have recently provided compelling evidence that the inclination toward spirituality is humanity’s default condition. Bloom (2007) argues that spirituality is akin to language, both occurring universally in humans, with universal deep structure, neither being present at birth, and both being shaped by culture. Bloom cites two lines of developmental evidence for the unlearned, innate nature of spirituality. The first concerns the apparently universal tendency of young children to implicitly endorse Cartesian dualism, typically assigning responsibility for rational cognitive tasks to the brain, and responsibility for the emotional, creative, and mindless events to a non-neural mind, soul, or inner self; this suggests that we are born with a default belief in the separability of bodies and souls, which opens the door to a range of spiritual beliefs. The second line of evidence concerns the human tendency, even in babies, to attribute human-like agency to a range of non-living entities and to attribute agency to nonrandom structures, opening the door to belief in an intentional designer. One perspective is that such tendencies are accidents of evolution, which upon coming into contact with inevitable problems of human insufficiency and tragedy, create the necessary and sufficient conditions for spirituality to prosper. An alternative perspective is that spirituality conveys survival benefits to human individuals and groups (e.g., in promoting altruism, justice, morality, etc.) and, hence, is a beneficiary of natural selection. Regardless of its specific evolutionary and developmental causes, spiritual motivations are real and they are powerful.

From a historical perspective, spiritual motivations have been a key driver of societal transformation, from bloody Crusades, Jihads, and Inquisitions to more prosocial movements. For the latter, one can take the example of the history of the United States, where historians have demonstrated the power of uniquely spiritual motivations in contributing to each great social revolution: the First Great Awakening (1730–1750), which fueled the American Revolution through its emphasis on liberty of conscience and freedom of religion (Kidd, 2009); the Second Great Awakening (1790–1840), which stoked abolitionist sentiment culminating in the American Civil War and the spiritually-inspired causes of freedom and piety that led to the Women’s Suffrage and Temperance movements in the early 20^th^ Century (Hankins, 2004), and the Civil Rights movement in the 1960s (Wallace, 2008). From a psychological perspective, spiritual motivations have been convincingly shown to exist separately from other motives, and to be uniquely predictive of a variety of important life outcomes, as will be reviewed below. Despite the central role of spirituality in so many people’s lives, academic social scientists have tended to ignore this domain in their research (Pargament, 2002; Pargament, et al., 2013). By overlooking this central life domain, marked by such strong, enduring, and life-shaping commitments, most psychological theories of motivation are incomplete. Indeed, Bloom (2007) has recently questioned why spirituality has been so neglected in the psychological literature. He dismisses as implausible the notion that spirituality might be of meager theoretical interest or practical relevance, citing the central role that it plays in people’s lives in shaping their behavior and socio-political views. He concludes that despite its “taboo” status within psychology, “any complete theory of human nature has to make sense of (spirituality)” (p. 147).

### Can Spiritual Motivation be Subsumed by Basic Motives?

Many social scientists have presumed that spiritual impulses can be explained by more fundamental psychological processes. In this view, spiritual sentiments are simply one more example from the plethora of culturally created categories that can be reduced to the actions of basic motives, such as the needs for affiliation or security. Motivation, here, will refer to processes that initiate and direct goal-oriented behavior in response to unmet needs (Pincus, 2006). Reiss (2004), for example, proposed that the 16 basic motivations postulated by his sensitivity theory can be used to understand the strivings^1^ that underlie spiritual behavior; he reports that the most common motive for spiritual behavior is fear of death, followed by other motives including strivings for interdependence, honor, loyalty, curiosity, order, status, vindication, acceptance, and so on. The attempt to reduce spiritual motivation to more mundane and basic elements is not new. In their survey of the history of spirituality as a motivational construct in psychology, Pargament et al., (2005) cite the early example of Leuba et al., (1934), who wrestled with the question of the existence of a spiritual instinct and concluded that mystical experiences could be fully explained using more basic physiological constructs. These authors note that Freud (2012) and Durkheim (1915) also saw spirituality as an expression of more basic underlying needs, for self-protective illusions and social unification, respectively. Karl Marx (1844) proposed that spirituality is a means of *symbolically* reuniting alienated workers with the natural means of production, and, as such, is a symptom of oppression. More recently, spiritual strivings have been attributed to a general need for control (Gibbs, 1994).

If spiritual impulses could be completely explained by the operation of basic motives, there would be no need for the postulation of distinctly spiritual strivings, no more so than a distinct motive for gardening would be needed to explain planting and weeding. However, there is a fundamental difference between explication of the motivations that can *contribute* to a person’s spiritual strivings (to have spiritual feelings and behave in accordance with spiritually-derived precepts) and to identify motivations that are *uniquely* associated with spirituality.

As is so often the case in psychology, Gordon Allport (Allport & Ross, 1967) has provided a key distinction that serves to distinguish uniquely spiritual motivations from those non-spiritual motives that may simultaneously contribute to spiritual thought and action. Allport differentiated between intrinsic and extrinsic spirituality, such that extrinsic spirituality represents the tendency to engage in the spiritual domain as a means to achieve desired goals, such as acceptance, security, or social esteem, whereas intrinsic spirituality denotes the desire to be spiritual for its own sake (Park & Edmondson, 2011). In Allport’s words, in contrast to an extrinsically motivated person who “*uses* his religion, the intrinsically motivated *lives* his… (finding his) master motive in religion” (Allport & Ross, 1967, p. 434; italics added). The notion that spiritual motivation can be fully explained by more basic motives inherently assumes that all spiritual motivation is ultimately *extrinsic*, that it always serves as a means to a more basic end. However, as Allport suspected, a great deal of evidence has accumulated that much spiritual motivation is indeed intrinsic and unique.

### The Uniqueness of Spiritual Motives

Following Pargament (1997; Pargament et al., 2005), spirituality can be defined as the search for significance in ways that are related to the *sacred*. Sacredness denotes things or concepts that are holy (i.e., qualitatively distinct from the non-holy or mundane) and worthy of veneration, and this process of sanctification can extend to material objects, time, space, life events, literature, music, people, ideas, social categories, and roles – anything that can be associated with the divine (Pargament et al., 2005). In its’ structuring of the sacred, spiritual systems are unique among human institutions and processes, and this unique focus is related to a variety of real-world outcomes.

Pargament et al. (2005) present empirical evidence of the unique explanatory power of spiritual indicators that substantially improve the predictability of a variety of important outcomes such as mortality, and both physical and mental health.

#### People are Aware of Their Uniquely Spiritual Strivings

When asked to list personal strivings, uniquely spiritual strivings tend to be spontaneously reported by 28% of community adults, and these strivings tend to supersede all others in terms of priority (Emmons, 1999). The presence of spiritual strivings is linked with lower levels of inter-goal conflict, suggesting that these strivings function as *master goals* that serve to integrate other goals (Emmons, 2005).

#### Spiritual Strivings Emerge as a Distinct Factor Among Personality Traits and Motivations

Measures of *spiritual transcendence* emerge as a distinct factor when measured alongside the big five personality factors (i.e., neuroticism, extraversion, openness, agreeableness, and conscientiousness), indicating that this tendency cannot be reduced to more basic personality factors (Piedmont, 1999).

#### Spiritual Strivings are Uniquely Linked to Multiple Indicators of Subjective Well-Being

Indicators of well-being (e.g., life satisfaction, absence of depression, marital satisfaction) are more strongly correlated with the presence of spiritual strivings than with any other type of striving, and that these relationships persist after controlling for more basic motivations, such as desires for affiliation and nurturance (Emmons, 1999). From a trait perspective, the spiritual personality factor has been shown to uniquely predict well-being (e.g., level of stress, social support) above and beyond other traits (Piedmont, 1999).

Spiritual strivings have been shown to make a significant and unique contribution to satisfaction with coping, reduced anxiety and depression, remission of depression, greater meaning and purpose in life, and perceptions of spiritual growth (Koenig et al., 1998; Mickley et al., 1998; Musick & Strulowitz, 1998). For example, spiritual coping has been shown to predict life satisfaction after kidney transplantation above and beyond measures of cognitive restructuring, sense of control, and social support (Tix & Frazier, 1998).

#### Spiritual Strivings are Uniquely Linked to Multiple Health Indicators

Spiritual strivings are linked to multiple indicators of physical health. These include morbidity, mortality, cardiovascular functioning, pain perception, and immune function, as well as compliance with doctor’s orders (George et al., 2002; Hummer et al., 1999; Koenig et al., 2012; McCullough et al., 2000). All have found evidence of direct effects of spiritual involvement on reduced morbidity and mortality beyond the effects mediated by social support and health practices.

#### Spiritual Strivings Behave as Motivations

A variety of authors have addressed the question of the extent to which spiritual strivings drive actual behavior. For many authors, this question seems self-evident:

“That religious beliefs (*i.e., those derived from formal spiritual systems)* motivate individuals, groups, and even whole societies is beyond doubt. In the name of religious beliefs people have gone to war and advocated peace, have hated and loved, have argued and been reconciled, have taken the lives of others and given their life for others, have been spurred to great achievement and have eschewed worldly achievement altogether. In fact, if evaluated in terms of the motivational intensity of behaviours associated (with) them, religious beliefs would appear to be among the most salient of human beliefs” (Dowson, 2005; p. 19).

Schnitker and Emmons (2013) note the work of Trout (1931), who recognized the teleonomic (purposeful and goal-directed) character of spiritual behavior, and Gordon Allport (1961) who suggested that spiritual behavior is distinctively intentional. The relative degree of correspondence between spiritual attitudes and prosocial behaviors, known as the “judgement/action issue,” has been extensively researched (see reviews in Batson et al., 2005 and Rosenkoetter, 2005), necessitating the incorporation of motivation as an explanatory construct. Martin Hoffman, in particular, has called for acknowledgment of the centrality of moral motivational-emotional processes in driving moral behavior; Allport (1961) similarly believed that moral thoughts lead to moral actions because of the motivating power of intrinsic spiritual meaning (Rosenkoetter, 2005).

To what extent can spiritual strivings be described in terms of the Expectations (being), Experiences (doing), and Outcomes (having) hierarchy? Schnitker and Emmons (2013) explicitly integrate a motivational perspective into the theory of spirituality, adopting goals theory to describe the goal-directed nature of spiritual strivings, specifically, “what a person is trying to do, be, or achieve in relation to religion.” Note that this trifold distinction of motivations existing at the level of “being,” “doing,” and “having” corresponds precisely to the modes of existence proposed by Forbes (2011) in their motivational taxonomy as the rows of Expectations (being), Experiences (doing) and Outcomes (having).

### How it Works: The Role of Meaning

The accumulated evidence suggests that spiritual strivings play an important and unique role in shaping other motives, behavior, and health, but through what mechanism? Current research suggests that spirituality operates by organizing *meaning*, both global meaning (e.g., the meaning of life) and situational appraisals (e.g., the meaning of an illness; Park, 2013). Indeed, research has demonstrated that spirituality is strongly associated with having a sense of meaning in life (Tomer & Eliason, 1996).

The development and presence of global meaning in a person’s life has been linked to both protective and growth outcomes. Viktor Frankl famously reported that those with a sense of their life’s purpose were far more likely to survive the Nazi concentration camps and went so far as to state that “man’s search for meaning is the primary motivation in his life, not a ‘secondary rationalization’ of instinctual drives” (Frankl, 1985, p. 121). Research in positive psychology has similarly concluded that authentic happiness can only be achieved through the confluence of pleasure in the moment and meaning, the connection to a larger truth that will influence the future (Ben-Shahar, 2007; Seligman, 2004).

The need for meaning has been proposed as the fundamental organizing principal for the psychology of spirituality, with implications for cognition and motivation (Hood et al., 2009; Schnitker & Emmons, 2013). The close link between spirituality and meaning exists because spiritual systems play a unique role in meaning making systems (Park, 2013). This dynamic was recognized by Kohlberg and Power (1981), who defined spirituality as the kind of “human reflection that imparts meaning and purpose to life” (Rosenkoetter, 2005). As recognized by the positive psychologists, spirituality is linked to authentic happiness (Bono et al., 2004) because it fuels meaning. In terms of its protective function, spirituality informs the process of making meaning out of life circumstances, which is an essential part of coping. In addition to the functions of spiritual meanings, the unique *content* of spiritual strivings adds to their power. Spiritual goals tend to transcend the self by addressing the ultimate meaning of existence (Emmons, 2005), providing believers with a master life plan. According to Pargament (1997), a key element that provides much of the power of spirituality to create meaning is the concept of the *sacred* as a “response to the problem of human insufficiency” (p. 310).

### Incorporating Spiritual Motivation Within a General Taxonomy of Motivation

The goal of this paper is to define a taxonomy of motivation that can accommodate spiritual motivations alongside non-spiritual motivations (e.g., Maslow’s need hierarchy, etc.) by extending the 9-cell taxonomy of human motivation proposed by Forbes (2011) into a 12-cell taxonomy. The 9-cell taxonomy is formed by crossing three domains of human life, the Intrapsychic (within the self), the Instrumental (within the material world), and the Interpersonal (within the social world) with three states of being, Expectations (forward looking), Experiences (in the moment), and Outcomes (backward looking). This paper proposes that this model should be extended by adding a fourth domain of human life, the Spiritual.

Simultaneous with the writing of the present article, Davila and Crawford (2018) proposed a very similar conceptual extension of the Forbes matrix as applied to understanding employee needs in the workplace, suggesting that the model would benefit from inclusion of *transcendental needs*, which they define as the “need to believe in something” related to the “realm of abstract ideas and spirituality.” Consistent with the present argument, these authors suggest that this category of needs pertain to *idealized outcomes* such as hope, renewal, transformation, and transcendence, and refer to the centrality of concepts of meaning, purpose, and the sacred in this domain.

There are, however, fundamental structural differences between the model proposed herein and the model proposed by Davila and Crawford (2018). The primary difference pertains to the location of the new spiritual/transcendental concept: Davila and Crawford (2018) add it as a fourth level of attainment (“row”), “idealized outcomes,” following the triad of expectations (being), experiences (doing), and outcomes (having). The present article proposes no change to the row structure describing the three levels of attainment, but instead suggests the addition of the Spiritual as a fourth life domain (“column”), after the domains of the Self, the Material, and the Social. A second difference between the models is the addition of a third dimension by Davila and Crawford (2018): In addition to Forbes’ (2011) dimensions of level of attainment (“rows”) and the life domain (“columns”), these authors add a dimension representing the type of need, with levels they define as *physiological*, *social*, and *transcendental*.

Davila and Crawford (2018) very helpfully present empirical evidence using measures of transcendental and non-transcendental needs alongside assessments of Big Five personality traits (i.e., openness, conscientiousness, extraversion, agreeableness, neuroticism) providing compelling support for the separate existence of spiritual/transcendental needs. These authors hypothesize that of the Big Five, only openness would be predicted by the level of spiritual/transcendental needs, and, indeed, reported that of the 28 correlations examined, the openness-transcendental need correlation (0.35, p < 0.001) was strongest and most statistically significant. Perhaps more importantly, this study demonstrated that spiritual/transcendental needs are not explainable by four of the Big Five personality traits, and that even openness could explain only a small fraction of its variance, a result consistent with those reported by Piedmont (1999). These results, considered in conjunction with the vast array of findings of the unique predictive ability of spiritual needs, strongly supports an empirical basis for concluding that such needs deserve to be incorporated into any integrative model of human motivation.

Beyond the empirical evidence, it is important to recognize the accumulated wisdom represented by global theoretical traditions that explicitly acknowledge the Spiritual as a fourth life domain.

We find an abundance of four-domain systems that incorporate an explicitly spiritual domain in widely disparate fields, including in all five of the major global religions (Judaism, Christianity, Islam, Buddhism, and Hinduism); philosophy of religion; motivational, developmental, and sports psychology; and, more popularly, in the area of personal growth (see Table 2).

**Table 2:** Thirteen theoretical systems that explicitly incorporate a spiritual life domain

Field	Source	Self	Material/ Physical	Social	Spiritual
Motivational Psychology	Forbes (2011) Taxonomy of Human Motivation	Intrapsychic	Instrumental	Social	< missing >
Developmental Psychology	William James’ (1890) Theory of Self (see Cooper, 1992)	Pure Ego	Material Self	Social Self	Spiritual Self
Developmental Psychology	Maslow’s Hierarchy of Needs	Self-Actualization	Physiological, Security	Belonging, Esteem	Self-Transcendence
Psychology of Religion	Fowler (1981) Stages of Faith (integration of models of Erikson and Kohlberg)	Intuitive-Projective & Mythic-Literal	Synthetic-Conventional & Individuative-Reflective	Conjunctive Faith	Universalizing Faith
Psychology of Religion	Peck (2010)	Chaotic/ Antisocial	Formal/ Institutional	Skeptical	Mystical
Philosophy of Religion	Fisher (2011) Four Domains Model	Personal	Environmental	Communal	Transcendental
Philosophy	Martin (1986) Four Sources of Fulfillment (cited in Covey, 1989)	Autonomy (mental)	Tone (physical)	Resonance (social connection)	Perspective (spiritual)
Sports Psychology	Sheehan (1992) Four Human Needs Addressed by Physical Activity (cited in Siegel, 1995)	Craftsman	Animal	Friend	Saint
Personal Growth	Covey (1989) Four Dimensions of Human Nature	Mental	Physical	Social/Emotional	Spiritual
Religion	Wolfson (2020) Judaism’s Four Levels of the Soul (cited in Miller, 2018)	Neshama, use of intellectual comprehension	Nefesh, awareness of physical body and physical world	Ruach, awareness of the socio-emotional	Chaya/Yechida, direct knowledge of, and unification with, the divine
Religion	Caschetta (2015) The Four Pillars of Catholicism	Faith in the Creed, within the individual	Sacraments, perceived through the senses	Morality, obeying commandments governing social relationships	Prayer, relationship & communion with the divine
Religion	Sahin (2003) Islam’s life domains	Nafs (self/ego)	Dunya (material world)	Suhba (social companionship)	Ruh (spirit)
Religion	Koller (1968) Hinduism’s Four Purusharthas (Human Objectives)	Kama (Personal pleasure)	Artha (Worldly success)	Dharma (Duty, primarily to community)	Moksha (Transcendent liberation from the cycle of death and rebirth)
Religion	Anderson (2013) Buddhism’s Four Noble Truths	Dukkha, craving and clinging to impermanent states and things	Samudaya, material craving produces karma (being trapped in dissatisfying cycle of rebirth)	Nirodha, beginning of cessation of cravings	Marga, the path leading to Nirvana (transcendence)

### A Fourth Spiritual Domain in Accumulated Human Wisdom

Each of the thirteen systems appearing in the rows (below the first row represented by Forbes, 2011) provide for a spiritual fourth life domain. In all cases, the spiritual domain is consistently assigned the last and highest position among life domains. Significantly, these systems typically present the life domains in the same order as Forbes (2011), namely, the Intrapersonal (within the self), the Instrumental (the material world), and the Interpersonal (the social world), but go on to add the Spiritual.

Within the psychology literature, William James’ (1890) Theory of Self posits the existence of Pure Ego (one’s perception of a unified self), upon which is built a Material Self (one’s desires for “adornment, foppery, acquisitiveness, constructiveness,” etc.), upon which is built a Social Self (one’s desires “to please, be noticed, admired, etc.”), upon which is finally built a Spiritual Self (one’s desires for “intellectual, moral and religious (*i.e., spiritual*) aspiration”; p. 329).

Within the philosophy literature, the models of spirituality proposed by Fowler (1981), Peck (2010), and Fisher (2011) follow the same expanding circles of concern, beginning with the self, then the material environment, then the social or communal, and finally, the spiritual (alternatively labeled the transcendental, mystical, or universal). Fowler’s work is particularly relevant as it explicitly employs the developmental stage models of both Kohlberg (1958) and Erikson (1963). The very same conceptual domains, in the same order, are posited by Martin (1986), Sheehan (1992), and Covey (1989).

The five major world religions, as the most ubiquitous formal spiritual systems, draw the same four distinctions in the same order. The Kabbalistic tradition within Judaism (Miller, 2018) posits that the soul develops from a starting point of *Neshama*, basic awareness of the self, to *Nefesh*, awareness of the material, to *Ruach*, awareness of the socio-emotional, and finally to *Chaya*, direct knowledge of the Divine, and *Yechida*, unification with the Divine. Caschetta’s (2015) Four Pillars of Catholicism starts with Faith in the Creed within the self, develops to adoption of material Sacraments, which then expands to social Morality, and finally to Prayer, i.e., communication & communion with the divine. Islam’s life domains begin with *Nafs*, the selfish ego that must be overcome, interaction with the material world represented by *Dunya* and the social world represented by *Suhba*, and finally, the spirit, *Ruh*, which is not bounded by the physical universe and is freed through communion with God (Sahin, 2003). Hinduism’s Four Purusharthas or human objectives follow the same logic starting with *Kama*, the pursuit of personal pleasure, then *Artha*, the pursuit of material success, then *Dharma*, the desire to perform one’s communal duties, and finally, *Moksha*, transcendence into the divine *Brahman* with liberation from *saṃsāra*, the cycle of death and rebirth (Koller, 1968). Lastly, Buddhism’s Four Noble Truths parallel Hinduism’s Purusharthas beginning with *Dukkha*, selfish cravings, followed by *Samudaya*, cravings of the material, which is the root cause of *karma*, being trapped in a dissatisfying cycle of rebirth, which is followed by *Nirodha*, the beginning of cessation of cravings, ultimately leading to *Marga*, the path leading to *Nirvana* or spiritual transcendence (Anderson, 2013).

### A Heuristic Model of Human Motivation

**Fig. 1:**
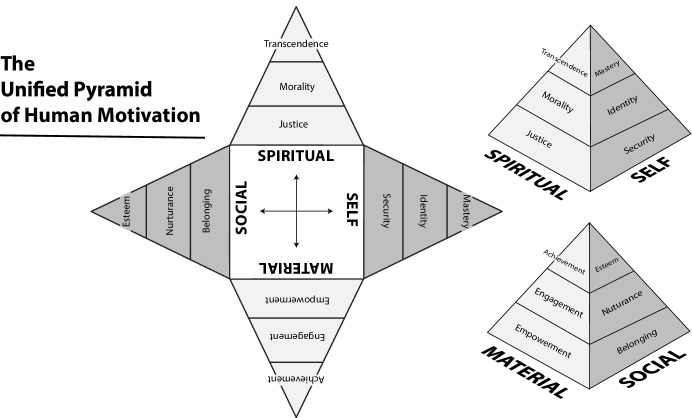
A unified pyramid of human motivation

Using both established theory and empirical evidence as a basis, it is proposed that the most parsimonious taxonomic model of human motivation would be represented by a four sided structure that adjacently links the domains of the Intrapsychic and Interpersonal domains to the Spiritual domain on one side, and linking the Intrapsychic and Interpersonal domains to the Instrumental domain on the side opposite the Spiritual. Two additional heuristic modifications are suggested to improve upon the Forbes (2011) matrix: Firstly, we propose that the direction of the rows should be flipped such that the “higher,” less commonly actualized motivations should appear at the top of the matrix, and the “lower” or more basic motivations should appear at the bottom of the matrix. Secondly, a pyramidal structure is suggested as a replacement for either a flat table or cube to reinforce the notion that humans must start from the basic motivations within each of the four domains before ascending to the salience of higher motivations; consequently, progressively fewer humans attain the higher levels with each domain, shrinking their relative sizes toward the top as visually represented by a pyramid (see Fig. 1).

### The Spiritual Triad

As noted above, the organizing rows of the Forbes (2011) matrix represent three different kinds of fulfillment that motivations can pursue, corresponding to three modes of existence: forward-looking expectations (ways of *being*), experiences in the present moment (ways of *doing*), and backward-looking outcomes (ways of *having*)*.* Forbes (2011) also suggests that movement from forward-looking levels within each domain to present-experience and to backward-looking levels is developmental, in effect, building on the satisfaction of lower levels to permit the activation of higher levels, respectively. We will now discuss the ways in which the proposed levels of the Spiritual domain are developmentally progressive.

The first level, forward-looking expectations (*being*), addresses “establishing and enhancing one’s potential and resources for action in life in the future” (p. 88). Motivations of this type focus on forward-looking potential and expectations in each of the life domains: In the domain of the Self, future growth is ensured through the establishment of psychological security and self-esteem (Security). In the Material domain, future growth is secured through the establishment of psychological empowerment and capability (Empowerment). In the Social domain, future growth is secured through establishment of basic social relationships and secure attachments (Belonging). The analogous type of motivation in the Spiritual domain would be the basic need for *justice and fairness*, which “secures the future” by defining standards of right and wrong, upon which moral and ethical guidelines can be built, which in turn, provide a scaffold for the desire for personal holiness and spiritual transcendence.

The second type, experiences in the present (*doing*), addresses desires to create “the best possible experiences in the moment” (p. 88). Motivations of this type, i.e., focused on present action, are manifest in each of the life domains: In the domain of the Self, this is represented by the need for self-expression and authenticity (Identity); in the Material domain, this is the need for immersive engagement (Engagement); in the Social domain, this is the need for caring and intimacy (Nurturance). The analogous type of motivation in the Spiritual domain would be the need to *adhere to moral principles and standards of ethics* in present behavior, built on the foundation of the need for justice, and supporting the next level of aspiration. The notion that the need for justice is a developmental precursor of the need for morality is explored below in the discussion of these two motives.

The third type, backward-looking outcomes (*having*), addresses “fulfillment from activity that has already taken place and is focused on the outcomes of that activity” (p. 88). Motivations of this type focus on evaluating the results of activity in each of the life domains: In the Self domain, past activities that fostered security and self-expression support the need for self-actualization (Mastery). In the Material domain, past empowerment and engagement supports the need for material Achievement. In the Social domain, past affiliations and loving relationships support the need for respect and admiration (Esteem). The analogous type of motivation in the Spiritual domain would be the need for *transcendence* of ordinary human limitations into the divine or universal, which rests upon the foundations of justice and adherence to moral and ethical guidelines. The notion that the need for morality is a developmental precursor of the need for transcendence is explored below.

Accordingly, based on the model’s structure, we hypothesize that stronger, more statistically significant positive correlations will exist between measures of the three levels of spiritual needs (the needs for justice, morality, and transcendence), than among these three needs and the other nine non-spiritual needs, and that substantially weaker or possibly negative correlations will exist between the three spiritual needs and the three material needs (the needs for empowerment, engagement, achievement).

Strong support for this proposition can be found in Kohlberg’s theory of moral development (1958), whose work was instrumental in organizing the spiritual triad. Kohlberg posited that moral reasoning develops through six stages, progressively becoming more sophisticated. Kohlberg believed that moral development was rooted in conceptions of justice, and that moral development continued throughout life. The six stages are grouped into three ascending levels of morality: pre-conventional, conventional, and post-conventional morality. Especially pertinent to the purposes of this paper, Kohlberg believed that the pre-conventional level was concerned with basic notions of justice; in the proposed triad, a *justice motive* is posited as the first building block of the spiritual domain. Kohlberg’s subsequent conventional and post-conventional stages of moral reasoning relate to motivation to maintain positive social relations and to uphold universal ethical principles; in the proposed triad, a corresponding *moral motive* is proposed as the intermediate level of spiritual motivation. Finally, Kohlberg proposed a seventh stage that he called *transcendental morality*, which he linked with spiritually-inspired moral reasoning; in the proposed triad, a corresponding *transcendental motive* toward unification with the universal or divine is proposed as the highest level of spiritual motivation.

#### Justice Motivation

The need to believe in a just world and a sensitivity to seek fairness.

At the basic level of building potential and expectations, in the Spiritual domain we find a key analogue of fundamental motivations of the other three life domains, i.e., Security, Empowerment, and Belonging. Justice is a key antecedent condition for personal security, since without justice no one, including one’s self, is safe. Justice also supports the foundations of belonging, providing each individual an opportunity to connect with others free from unjust social barriers such as racism, sexism, classism, etc. When a person is motivated by the need for justice, they seek procedural fairness, distributional fairness, and/or freedom from injustice. Justice indirectly provides the basis for personal empowerment, providing a secure platform for one to fairly build and express their potential, as in a fair meritocracy. Thus, we propose that justice motivation is strongly linked to Security motivation within the Intrapsychic domain, and to Belonging motivation within the Social domain, and less directly linked, and even potentially contrary, to Empowerment motivation. Accordingly, it is hypothesized that stronger, more statistically significant positive correlations will exist between measures of the need for justice and the needs for belonging and security, and that weaker or possibly negative correlations will exist between the need for justice and the need for material empowerment.

Starting at a very early age, humans are keenly aware of justice and fairness in circumstances affecting themselves and others. Hamlin et al. (2007) and Bloom (2010) have convincingly demonstrated in multiple experiments that even 8 month old babies possess a sense of justice, differentially preferring to look at characters who act benevolently, avoid looking at characters who do wrong, and prefer to look at characters who punish wrongdoers. Lerner’s Just World Hypothesis (1980; 2003) represents another empirically-supported theory positing widespread belief in the fundamental operation of justice in the world. In this way, belief in a just world represents a basic, at times primitive, and utilitarian motivation—it preserves the notion that a "contract" of sorts exists with the universe regarding the consequences of behavior, providing a safe foundation against which humans can plan and engage in activity.

#### Moral Motivation

The need to act in a manner that is in accord with moral principles specifying what is good and right.

Next in the triad of motives in the spiritual sphere is moral motivation. Atop the platform of basic differentiation between right and wrong needed for a sense of justice or fairness, the motive to perform good deeds involves striving to align one’s behavior with pre-ordained ethical standards or one’s conception of what is right. In support of this contention, evidence from neuroscientific fMRI studies (Robertson et al., 2007; Yoder & Decety, 2014; Decety & Yoder, 2017) demonstrates that sensitivity to injustice is predictive of neural responses to moral evaluations of others’ behavior, suggesting that an established sense of justice is prerequisite to the development of moral motivation.

The motive to perform good deeds reflects societal and spiritual norms as well as one’s personal attitudes and values of what is good and right. Like the justice motive, the need to perform good deeds as a primary moral motivation is also well established in the literature beginning with Immanuel Kant, who posited that the fundamental principle of morality is an objective, rational standard (the “Categorical Imperative”). In Kant’s formulation (Kant and Paton, 1964), this standard is an objective, necessary and unconditional principle to which we must adhere despite competing momentary desires. Specific moral requirements are justified by this rational standard; hence, immoral actions are necessarily irrational because they violate this standard.

Prentice et al. (2019) present evidence across four studies that moral motivation should be included as one of the basic human needs. Consistent with our definition, these researchers define moral needs as including desires for: (1) a strong sense of moral fulfillment, (2) being a good person, (3) embodying moral values, and/or (4) doing the right thing. Employing the criteria used by Sheldon, Elliot, Kim, and Kasser (2001) for determining if a motive should be considered a fundamental human need, they report that moral needs performed on par with Social Determination Theory’s (SDT) basic psychological needs of autonomy, competence, and relatedness in terms of directing cognitive processing, generating affective consequences, contributing uniquely to the prediction of well-being, and having enduring and prolific effects on psychological functioning. The work of Martela and Ryan (2020) that identifies “beneficence satisfaction and frustration” as a psychological construct distinct from SDT’s autonomy, competence, and relatedness similarly supports the inclusion of moral motivation as a fundamental need. They define beneficence as an act of charity, mercy, and kindness with a strong connotation of doing good to others, including moral obligation, and is therefore well-aligned with the present conceptualization.

Moral motivation is a central focus of the literature on altruism and prosocial behavior. Batson et al. (2007) identify four potential goals that can result in prosocial behavior: to benefit oneself (egoism), to benefit another person (altruism), to benefit a group (collectivism) and to uphold a moral principle (principlism or moral motivation). Because the proposed moral motive must reflect a desire to adhere to moral standards, it can reflect the operation of any of the latter three (altruism, collectivism, or principlism) to the extent that the behavior is sincerely motivated for the benefit of the other, the group, and/or the moral principle itself, but not to obtain benefit for oneself. Batson et al. (2003) are especially careful to distinguish between moral motivation, where the goal is to act in accord with moral principles, and altruistic motivation, where the goal is to increase another’s welfare. The extensive experimental literature on moral motives and empathy-induced altruism provides strong support for the existence of distinct subtypes of moral motivation. In particular, Batson et al.’s (2005) *religion as quest* motivation represents a strong exemplar of Spiritual domain motivation as it is associated with high levels of altruism that are highly responsive to the needs of suffering individuals and relatively insensitive to the presence of either personal or social rewards for the helper. Because it represents the “ultimate goal of upholding a moral principle” (p. 177), we suggest that it should be considered a pure form of moral motivation.

Evolutionary psychologists have tended to view altruism and moral motivation as the sequelae of innate, evolutionarily-adaptive social regulatory functions (Silk & House, 2011). Because both human and nonhuman primates rely heavily on social bonds for survival, the instincts underlying altruistic and moral motivations can be seen as products of evolution. To the extent that altruism promotes group acceptance, it can be seen as a means of securing access to social support, which is associated with both physical and mental health, and lower mortality, in both human and nonhuman mammals.

Staub’s (2005) motivational concepts of *moral courage* and *prosocial value orientation* also relate to the present definition of moral motivation. Similar to Batson et al., Staub views “motivation as moral when to some substantial degree its focus is to fulfill or live up to a moral belief, value, or principle” (p. 35), which he distinguishes from altruistic motivation, which is more “directly focused on the person rather than on a belief or principle.” Staub explicitly connects the development of these forms of motivation to the degree of nurturance experienced in childhood (Interpersonal/Social domain) and to the formation of a positive self-identity (Intrapsychic/Self domain), supporting the model’s structure proposed herein of closer connection of the Spiritual domain to the Intrapsychic/Self and Interpersonal/Social domains than to the Instrumental/Material domain. Accordingly, it is hypothesized that stronger, more statistically significant positive correlations will exist between measures of the need for morality and the needs for identity and nurturance, and that weaker or possibly negative correlations will exist between the need for morality and the need for material engagement.

#### Transcendental Motivation

The need to transcend one’s mind and body into the universal or divine.

The final member of the spiritual triad is transcendental motivation, which is stands atop the platform of moral motivation and action. This striving, toward transcending the here and now and the limitations of one’s material being, includes aspirations for unification or communion with the infinite and universal divine, achieving oneness. Intriguingly, an evolutionary function for transcendence motivation has recently been proposed by Price (2019).

The existence of a transcendental state of reality, i.e., a pure reality unconstrained by perceptual limitations imposed by space and time, was first proposed in the philosophy literature by Immanuel Kant (2003). This Kantian notion has been applied to a diverse set of issues in psychology including psychoanalysis (Stolorow, 2005), memory (Arcaya, 1991), and imagination (Waxman, 1991), among others.

The notion that the presence of moral motivation is a necessary prerequisite to the development of transcendental motivation is suggested in classic works by Jonathan Edwards (1741) and James (1985). Edwards, the most prominent figure of the First Great Awakening in the United States, specifies that the authenticity of a spiritual revival can only be judged by the ameliorative effects brought about in moral thoughts and actions. James (1985) echoes Edwards in stating that truly spiritual motivation can only be judged by its “moral helpfulness”; in other words, if it produces no positive moral effects, it can’t be spiritually significant, suggesting that morality is a necessary precondition for transcendence. James goes further by stating that transcendental motivation represents a next-level beyond moral motivation:

“Morality pure and simple accepts the law of the whole which it finds reigning, so far as to acknowledge and obey it, but it may obey it with the heaviest and coldest heart, and never cease to feel it as a yoke. But for religion (*as formal spiritual system)*, in its strong and fully developed manifestations, the service of the highest never is felt as a yoke. Dull submission is left far behind, and a mood of welcome, which may fill any place on the scale between cheerful serenity and enthusiastic gladness, has taken its place” (p. 46).

Due to the unfortunate heuristic convention of capping Maslow’s need hierarchy at the level of self-actualization, few students of motivation are aware that Maslow’s (1972) model actually contains a level beyond actualization, which he termed “self-transcendence”:

“Transcendence refers to the very highest and most inclusive or holistic levels of human consciousness, behaving and relating, as ends rather than means, to oneself, to significant others, to human beings in general, to other species, to nature, and to the cosmos” (p.279).

Because Maslow’s system is hierarchical, it, too, requires the satisfaction of all prior motivations before transcendence can be realized.

Transcendental motivation has traditionally been the purview of religions as formal spiritual systems. In Buddhism and Hinduism, *Nirvana,* literally “blown out” (i.e., the self is blown out like a candle), represents the transcendent condition beyond worldly suffering and desire, beyond even having a sense of self, wherein humans are released from both karma (the sum of a human's actions in this and prior states of existence, which determines one’s fate in future states of existence) and samsara (the cycle of death and rebirth), representing the highest and final human goal.

Within the Kabbalistic tradition of Judaism, the final and highest levels of personal attainment are *Chaya* (direct consciousness of the divine life force) and *Yechida* (literally, “oneness” or full unification with the divine), representing the very highest level of understanding attained by only rare individuals. Significantly, these states of existence are external to the body, underscoring their transcendent nature.

The term *communion* itself is strongly associated in Western cultures with Christianity. *Communion* is the ceremonial act by which the divinely instantiated *Eucharist* is shared and consumed, wherein “worshipers share bread and wine in the Eucharist as a sign of their unity with each other and with Jesus” (Oxford English Dictionary, 2018). This act of unification is further elaborated by Richstatter (2000), emphasizing that it represents both a symbol and source of unity:

“All who participate in this Eucharist are fed by the same life of Christ. At the same time, the worldwide eucharistic celebration is a sign of unity, it is also a source, or cause, of unity. We are nourished by the same body and blood of Christ, strengthened in unity (p.1).”

Transcendental motivation has been recognized by leading theorists in developmental and motivational psychology. As suggested at the start of this section, Kohlberg’s proposed 7^th^ stage of moral reasoning addresses the question “Why be moral?” in a manner that is inaccessible to earlier stages, which are limited to rational modes, but instead relies on "cosmic" or "infinite" perspectives. Significantly, these perspectives are rooted in “contemplative experience of non-egoistic or non-dualistic variety,” suggesting that these experiences extend beyond the self, material, and social worlds to the eternal and holistic. Kohlberg describes this motivation as “being a part of the whole of life and the adoption of a cosmic, as opposed to a universal humanistic …perspective” (Kohlberg, 1973, pp. 55- 56). Kohlberg and Power (1981) refer to the canon of natural law theory, citing Socrates, Martin Luther King, Jr., Marcus Aurelius, and Spinoza, as earlier attempts to address this cosmic perspective, as well as quantum mechanics for evidence of the "inseparable quantum interconnectedness of the whole universe" into an "unbroken wholeness." Spinoza’s theory of mind is particularly apt in this regard, summarized by Matson as "knowledge of the union that the mind has with the whole of nature is the true and highest good" (Matson, 1971). The motivation to experience this transcendent truth is the cognitive and affective component of what we mean by transcendental motivation, with the motivation for true unification with the divine representing the spiritual component.

In his work on prosocial behavior, Staub (2005) explicitly proposes that transcendental motivation can only come into play when lower needs are fulfilled: “When other needs are fulfilled, the need for transcendence, to go beyond the self, emerges or becomes more dominant. Thus, people whose basic needs have been constructively fulfilled are able to focus less on themselves and more on other people, the world, and spiritual matters” (p. 64). Staub suggests that the need for transcendence is satisfied by connecting one’s self “to nature or to spiritual entities” and by “helping others” (p. 38), suggesting, again, a strong linkage to the Social domain, particularly with Esteem motivation, the desire to set an admirable example for others. Achieving transcendence and unification with the divine, with deep respect given to other people, and indeed to all life, sets a strong social example.

As a final and highest state of perfection, the need to achieve transcendence should also be strongly linked to Mastery motivation, the quest for personal excellence that represents the highest level of the Intrapsychic domain. In contrast, transcendental motivation should not be directly connected to Achievement motivation, which is marked by the pursuit of material successes, the antithesis of transcendence. Accordingly, based on the model’s structure, we hypothesize that stronger, more statistically significant positive correlations will exist between measures of the need for transcendence and the needs for mastery and esteem, and that weaker or possibly negative correlations will exist between the need for transcendence and the need for material achievement.

## Discussion

### Structural Considerations

A model of human motivation is proposed that takes the form of a pyramid formed by four sides representing four life domains: the Self, the Material, the Social, and the Spiritual. By placing these domains as opposing pairs, Self vs. Social and Material vs. Spiritual, we are suggesting strong linkages between adjacent domains (e.g., Self – Spiritual – Social), and weak linkages for non-adjacent, opposing domains. We have indicated theoretical bases of support for this contention above in the work of Kohlberg and Power (1981), Staub (2005), and Schwartz (1996), however, empirical support also exists. The findings of Mahoney et al., (2005) on the probability of sanctification of life goals are strongly supportive of the proposed model, particularly with regard to the position of the spiritual domain relative to the other three domains. After explicitly spiritual goals, the personal strivings that are most likely to be sanctified (i.e., perceived to represent manifestations of God or exhibit other sacred qualities) tend to be those associated with the Interpersonal domain (i.e., helping others, family connections) and the Intrapsychic domain (i.e., existential issues and other self-relevant issues). Within each domain they found sanctification associated with certain types of social goals, such as altruism, and with certain self-oriented goals, such as existential goals, but not others. Social goals that are more distal (e.g., staying in contact with friends regardless of distance) as well as Intrapsychic self-improvement goals that blur into the Instrumental domain (e.g., to keep on learning and pursuing my degree) were less likely to be sanctified. Purely Instrumental goals, those related to the physical, material world (i.e., work, money, exercise, travel, home improvement, etc.) were *least* likely to be sanctified. These findings suggest strong linkage between the Spiritual domain and both the Interpersonal/Social domain and the Intrapsychic/Self domain, but not to the Instrumental/Material domain, resulting in the four hypotheses specified above.

Following Mahoney et al. (2005), degrees of sanctification can be thought of as a continuum, with certain life goals being highly likely to be sanctified, and others less likely. Such a continuum may be applied to the specific motivations within the Forbes (2011) life domains:

Within the Intrapsychic domain, the Security motive can involve minimal spiritual meaning in the case of physical safety or personal comfort, or it can involve a very high degree of spiritual meaning in the case of, for example, the “peace of knowing God.” Applying this distinction to the Identity motive, the striving to express one’s individual style may typically have no spiritual meaning, whereas the desire to express one’s individuality by living a simple ascetic lifestyle may have enormous spiritual meaning, as in the case of the distinctive dress of religious orders. Applying this distinction to the Mastery motive, the striving to achieve mastery of an art form may be an end in itself with no spiritual connotations, or it may be deeply suffused with spiritual meaning, as exemplified by J.S. Bach who famously stated that “the aim and final end of all music should be none other than the glory of God and the refreshment of the soul.”

Within the Social domain, the Belonging motive can have little spiritual significance as in the case of attending a college party, or it can have enormous spiritual significance in the case of a communal spiritual celebration. Applied to the Nurturance motive, striving to care for one’s pet may reflect substantial feeling of love but little spirituality; alternatively, caring for a dying parent often takes on significant spiritual meaning. Applied to the Esteem motive, the striving to receive acknowledgement from one’s peers may have no spiritual connotations, whereas setting a good example by working in a war zone with *Doctors Without Borders* may take on tremendous spiritual meaning.

But what of the Instrumental domain? To trace the origins of the antagonistic relationship between the Instrumental and Spiritual domains we must invoke the concept of *dualism*, the presumed separation of the material self (body and brain) from the immaterial (soul or spirit), versus *monism*, the presumed unity and reduce-ability of human nature (VanderStoep & Norris, 2005). Viewing material pursuits as being diametrically opposed to spiritual strivings is common to many formal spiritual systems but is perhaps most fundamental to the beliefs of Gnosticism and Manicheanism, which held that the material world is inherently evil, and that the non-material spiritual world is inherently good. Material-spiritual dualism is also prominent in Buddhism and Hinduism, where the highest levels of spiritual attainment necessitate the disavowal of and separation from the material world. There is abundant cultural and experimental precedent suggesting that Instrumental/material goals tend to be, at the very least, inconsistent with Spiritual goals. Within mainstream psychology, the same dualism can be found in Schwartz’s (1996) circumplex model of cultural values, which places strivings for material goals of achievement and power directly opposite transcendent goals of universalism and benevolence (Feather, 2005).

Following Allport’s distinction between extrinsic and intrinsic spiritual motivations, and the further elaboration of intrinsic *religion as quest* (Batson et al., 2005), we suggest that individuals marked by high *extrinsic* spiritual motivation use their spirituality for instrumental ends, and therefore, their observance reflects Instrumental motivation. In contrast to such instrumental-extrinsic spirituality, Batson et al. report compelling evidence that individuals with high intrinsic *religion as quest* motivation are more likely to engage in actual helping behaviors that support both self (Intrapsychic) and social (Interpersonal) rewards, and, accordingly, are more likely to consider themselves to be compassionate and caring. These findings support the proposed model’s designation of the Spiritual and Instrumental domains as polar opposites, as well as the linkage of the Spiritual to the Intrapsychic and Interpersonal domains.

Another feature of Forbes’ (2011) model of human motivation is the requirement that each motivation must be capable of operating as either a striving toward positive aspiration (i.e., promotion) or away from negative frustration (i.e., prevention). Each of the three motives proposed for the spiritual domain fulfill this requirement, i.e., one can seek ever higher conditions of justice, morality, or transcendence and one can be motivated by the presence of injustice, immorality, or the failure to transcend materiality. Emmons and Schnitker (2013) address this issue in their literature review, noting that spiritual strivings can take the forms of either approach goals or avoidance goals (e.g., “get closer to God” vs. “avoid God’s displeasure”), which are predictive of higher and lower well-being, respectively. Dowson (2005) suggests that the interaction of one’s personal and communal spiritual beliefs results in varying salience of approach versus avoidance motivations, e.g., focusing on positive spiritual aspirations vs. a “fire and brimstone” fear of going to Hell. Jackson and Francis (2004) bring this question to the neural level with findings suggesting that spiritual behavior engages the joint operation of the Behavioral Activation System (reward-seeking) and Behavioral Inhibition System (anxiety-avoiding). In this way, spiritual motivation adheres to the same Promotion-Prevention polarity as do all other motives in the model. Consistent with the Forbes matrix, it is hypothesized that measures of positive aspiration (e.g., I actively seek to become one with the universe) and measures of frustration avoidance (e.g., I wish I made more time to visit the sick) will emerge as separate subcomponents of each of the twelve distinct motivations specified by the model.

## Conclusion

The spiritual domain is central to the lives of most humans, and there is evidence that this has been the case since the pre-historic hunting and foraging days of our species (Harari, 2014). Formal spiritual systems provide many with their ultimate, highest-level goals, as well as guidance for achieving those goals. These goals can be described in terms of the three proposed motivations: Goals such as mercy, benevolence, compassion, generosity and forgiveness all speak to operations of the justice motive at the basic level of the spiritual domain. Goals such as righteousness, purity, nobility, and virtue all speak to operations of moral motivation, representing the intermediate level of the spiritual triad. And goals such as salvation, enlightenment, sensing God’s presence, and achieving Nirvana all speak to the operations of transcendental motivation.

Despite the many attempts of social scientists to reduce spiritual motivation to more basic motives, such motives remain stubbornly independent of other constructs. Recent work in evolutionary and developmental psychology has demonstrated the existence of innate “default” settings in humans that predispose them to spiritual strivings. Spiritual strivings are phenomenologically experienced and objectively powerful, serving as master goals that organize lower-order goals; they emerge as distinct factors when studying personality; and they are uniquely predictive of subjective well-being and health. There is substantial evidence that the power of spiritual motives may be due to their unique ability to provide life meaning and purpose in the face of human insufficiency. Only by formally incorporating the spiritual domain, with its three distinct motives, into a model and taxonomy of motivation can it be worthy of the title “unified model of human motivation.”

### Future Directions

We propose a series of five testable hypotheses that, if supported, will contribute substantial validation to our model:

- H1. Stronger, more statistically significant positive multiple correlations will exist between measures of the three spiritual needs (the needs for justice, morality, and transcendence), than among these three needs and the other nine non-spiritual needs, and that substantially weaker or possibly negative multiple correlations will exist between the three spiritual needs and the three material needs (the needs for empowerment, engagement, achievement).
- H2. Stronger, more statistically significant positive correlations will exist between measures of the need for justice and the needs for belonging and security, and that weaker or possibly negative correlations will exist between the need for justice and the need for material empowerment.
- H3. Stronger, more statistically significant positive correlations will exist between measures of the need for morality and the needs for identity and nurturance, and that weaker or possibly negative correlations will exist between the need for morality and the need for material engagement.
- H4. Stronger, more statistically significant positive correlations will exist between measures of the need for transcendence and the needs for mastery and esteem, and that weaker or possibly negative correlations will exist between the need for transcendence and the need for material achievement.

## Footnotes

1 We use the term *strivings* as shorthand for unmet psychological needs that elicit motivation toward fulfillment.
