# Correction to: Theoretical and Empirical Foundations for a Unifed Pyramid of Human Motivation

**DOI:** 10.1007/s12124-022-09709-0

**Published:** 2022-06-17

**Authors:** J. David Pincus

**Affiliations:** 1 Employee Beneft Research Institute, Washington, DC USA; 2 Research and Development Department, Leading Indicator Systems, One Franklin Street, Boston, MA 02110 USA

**Correction to** **:** **Integrative Psychological and Behavioral Science**

**https://doi.org/10.1007/s12124-022-09700-9**

The original version of this article unfortunately contained a mistake in Fig. 1. Below is the correct Fig. 1.
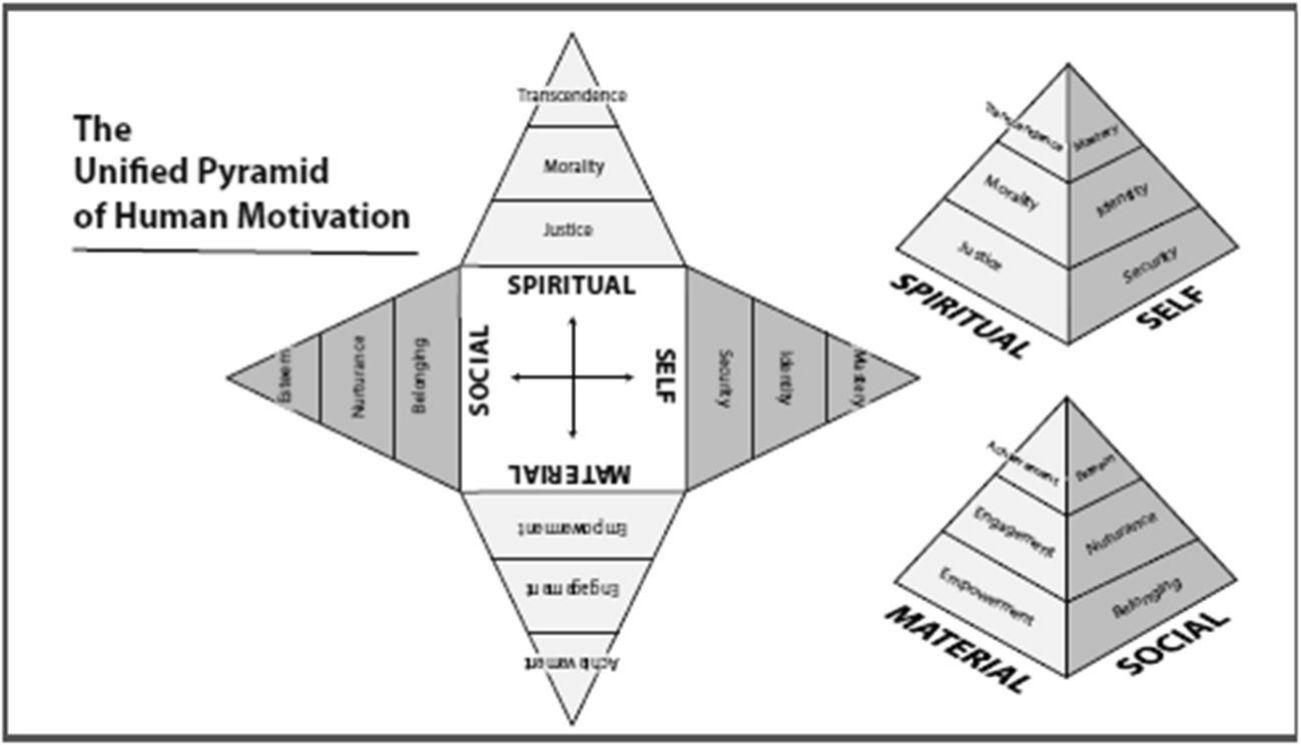


The original article has been corrected.

